# Acute effect of a supplemented milk drink on bone metabolism in healthy postmenopausal women is influenced by the metabolic syndrome

**DOI:** 10.1186/s12937-015-0092-2

**Published:** 2015-09-25

**Authors:** Sunethra D. C. Thomas, Howard A. Morris, B. E. C. Nordin

**Affiliations:** 1 Department of Medicine, University of Adelaide, Adelaide, South Australia Australia; 2 Endocrine and Metabolic Unit, Royal Adelaide Hospital, Level 7, Royal Adelaide Hospital, North Terrace, Adelaide, South Australia 5000 Australia; 3 School of Pharmacy and Health Sciences, University of South Australia, Adelaide, South Australia Australia; 4 Chemical Pathology, SA Pathology, Adelaide, South Australia Australia

**Keywords:** Dietary calcium, Postmenopausal, Metabolic syndrome, Osteoporosis

## Abstract

**Background:**

Dietary factors acutely influence the rate of bone resorption, as demonstrated by changes in serum bone resorption markers. Dietary calcium exerts its effect by reducing parathyroid hormone levels while other components induce gut incretin hormones both of which reduce bone resorption markers. The impact of dietary calcium on bone turnover when energy metabolism is modulated such as in metabolic syndrome has not been explored. This study was designed investigate whether metabolic syndrome or a greater amount of visceral fat influences the impact of dietary calcium on bone turnover.

**Methods:**

The influence of the metabolic syndrome on effects of dietary calcium on bone turnover in community dwelling postmenopausal women was studied. Twenty five volunteers consumed 200 mL of low fat milk with additional 560 mg calcium (one serve of Milo®) in the evening on one occasion. Fasting morning serum biochemistry before and after the milk drink with lumber spine bone density, bone mineral content, fat and lean mass using dual energy X-ray absorptiometry (DXA) and waist circumference were measured. The women were divided into 2 groups using the waist measurement of 88 cm, as a criterion of metabolic syndrome. Student’s t tests were used to determine significant differences between the 2 groups.

**Results:**

The lumbar spine mineral content was higher in women with metabolic syndrome. After consuming the milk drink, serum bone resorption marker C terminal telopeptide (CTX) was suppressed to a significant extent in women with metabolic syndrome compared to those without.

**Conclusions:**

The results suggests that dietary calcium may exert a greater suppression of bone resorption in post-menopausal women with metabolic syndrome than healthy women. Despite substantial evidence for close links between energy metabolism and bone metabolism this is the first report suggesting visceral fat or metabolic syndrome may influence the effects of dietary calcium on bone metabolism.

## Introduction

Osteoporosis is common in postmenopausal women, in which estrogen deficiency plays an important role. Low dietary calcium intake and vitamin D deficiency also contribute to the high prevalence of osteoporosis amongst these women. Post-menopausal bone loss is driven by increased rate of bone resorption relative to bone formation which is demonstrated biochemically by an increase in serum levels of metabolites of type I collagen.

Dietary calcium and vitamin D are essential for skeletal health throughout life and are recognised as bone sparing nutrients [1, 2]. The calcium requirement rises after the menopause due to increased losses and reduced intestinal absorption [3, 4].

The main dietary source of calcium is dairy food with high calcium bioavailability and optimal ratio of calcium to phosphate optimal for intestinal absorption [5].

Regular intake of vitamin D-fortified, calcium rich food, such as dairy inhibits markers of bone resorption in postmenopausal women -within 4 to 8 weeks [6–8]. An inadequate dietary calcium intake in post-menopausal women leads to increased risk of osteoporosis and fragility fractures [9]. However a longitudinal prospective cohort study did not show a reduction in fracture risk or osteoporosis rates with an increased dietary calcium intake [10].

The variable results in terms of bone mineral density and fracture reduction may arise from factors other than dietary calcium and vitamin D influencing bone metabolism. Among these factors, energy nutrients as well as dysregulation of metabolic pathways as occurs in metabolic syndrome may be important factors.

To our knowledge, the acute effects of dietary calcium on bone metabolism in the context of metabolic syndrome in postmenopausal women have not been evaluated. The present study determined the interaction between a single evening supplemented milk drink and anthropormetric and biochemical measures of metabolic syndrome on bone resorption in post-menopausal women. The aim is to investigate if metabolic syndrome or a greater amount of visceral fat influences the impact of dietary calcium on bone resorption in post-menopausal women.

## Method

### Participant recruitment

Ethics approval as granted by the Royal Adelaide Human Research Ethics Committee (Protocol number 09071; 29th July 2009). Community dwelling postmenopausal women who participated in a non interventional observational study were invited to participate in the current study. They completed a general health, a dietary calcium intake questionnaire and a consent form. Subjects who were taking calcium supplements were instructed to cease the supplements for 7 days prior to participation.

### Intervention

On the day of the study each subject had morning fasting blood samples collected for routine electrolytes, liver function, creatinine, urea, total cholesterol, glucose, calcium, phosphate (analysed by Olympus AU 5700, Olympus Japan), parathyroid hormone (PTH,—Immulite 2000, Siemens Healthcare Australia), 25OH vitamin D (25OHD) (IDS-iSYS, Immunodiagnostic Systems, −UK) and C-terminal telopeptide of type I collagen (CTX,—Roche E170, Roche Diagnostics, Australia). Subjects were provided with Pura Tone milk (Pura, Victoria, Australia) (1 L) and a sachet of Milo (Nestle, Australia) (20 g) with a 200 mL plastic measuring cup. They were instructed to measure out 200 mL milk (containing 400 mg Ca, 10 g protein, 13.5 g carbohydrate) and add the sachet of Milo (containing 160 mg Ca, 2.6 g protein, 13.3 g carbohydrate). The drink (containing a total of 560 mg Ca) either warm or cold, was taken at 9 pm the same night as the blood test. The next morning another set of fasting blood samples were collected from each subject to repeat the assays of the biochemical variables, except for 25OHD. All subjects had a radiocalcium absorption study performed using ^45^Ca [11]) within 12 months of the study and a DXA bone mineral density measurement (Norland XR 36, Swissray International Switzerland) within 6 months of the study. An abdominal fat and lean mass obtained from the lumbar spine DXA, height, weight and waist circumference (WC) were recorded. Subjects—completed a validated food frequency questionnaire to ascertain their habitual dietary calcium intake.

Subjects were divided into 2 groups according to their WC using 88 cm as a cut off with WC > 88 cm indicating metabolic syndrome (ATP III criteria) [12]. WC was chosen to categorise subjects into metabolic syndrome because it compares closely with the BMI and visceral fat. Waist circumference is also the theme of a public education campaign conducted by the Heart Foundation of Australia [13]. The Heart Foundation recommends measuring the waist circumference as an inexpensive and non-invasive method to indicate visceral fat and a predictor of risk for chronic disease such as diabetes and heart disease.

### Statistical analysis

A Student’s *t* test was performed to determine significant difference between measured variables before and after the supplemented milk drink, and also differences between the metabolic syndrome women and lean women. Linear regression analysis and a correlation matrix were used to determine correlations between variables.

## Results

Twenty five postmenopausal women volunteered to participate in the study. Demographic data and anthropometric measurements are given in Table 1.

**Table 1:** Demographic and anthropometric data for all subjects

Variable	Mean	SD	Range
Age (years)	63	5	51–71
Years since menopause	12	7	1–28
Height (m)	1.62	0.05	1.55–1.71
Weight (kg)	66	9	50–81
Waist circumference (cm)	84	9	67–99
BMI (kg/m2)	24.9	3	20–31
Habitual daily Ca intake (dietary; mg)	1012	260	642–1605

Their mean fractional calcium absorption was 0.67 (range 0.33 to 1.24; normal >0.5) and mean urine calcium/creatinine ratio was 0.28 (range 0.03 to 0.73; reference interval 0.03–0.4). There was no significant difference in the fractional calcium absorption, urinary calcium excretion or habitual daily calcium intake between the women with and without metabolic syndrome, as defined by a WC > 88 cm.

**Fig. 1:**
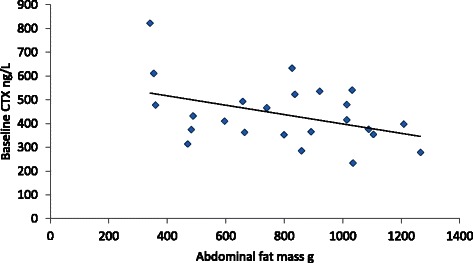
Visceral fat mass is significantly inversely related to baseline fasting CTX in all subjects (R^2^ = 0.18; *P* = 0.04)

Serum CTX levels, a marker of bone resorption, was negatively related to abdominal fat mass (Fig. 1) and serum 25OHD was negatively related to WC (*P* < 0.01) amongst all the subjects at baseline. Biochemical variables before and after the supplemented milk drink for all women are presented in Table 2. Only ionised calcium changed significantly after the supplemented milk.

**Table 2:** Biochemical variables before and after the supplemented milk drink

Variable	Before drink [mean (SD)]	After drink [mean (SD)]	Level of significance
Glucose mmol/L	4.9 (1)	4.8 (1)	0.4
Total cholesterol mmol/L	5.6 (1)	5.6 (1)	0.4
Total Ca mmol/L	2.37 (0.08)	2.4 (0.09)	0.12
Ionised Ca mmol/L	1.21 (0.04)	1.23 (0.04)	0.05*
Phosphate mmol/L	1.18 (0.13)	1.22 (0.13)	0.15
ALP IU/L	75 (19)	71 (20)	0.24
CTX ng/L	432 (132)	416 (143)	0.3
PTH pmol/L	4.3 (1.1)	4 (1)	0.13
25 OHD nmol/L	88 (26)	NA	NA

*P = 0.05

WC, fasting blood glucose and lipid profiles were available as determinants of metabolic syndrome in these women. Fasting blood glucose of >5.6 mmol/L, triglyceride >1.7 mmol/L and HDL < 1.29 mmol/L (as suggested by the American Heart Foundation) [14] were not used because these biochemical criteria did not distinguish between the 2 groups. In support of the use of WC as a marker of metabolic syndrome the change in blood glucose after the supplemented milk drink correlated with WC, abdominal fat mass and body weight (*P* < 0.01).

Table 3 represents the anthropomorphic measures on the two groups based on WC (<88 cm compared with > 88 cm). There was no significant difference in the fractional calcium absorption, urinary calcium excretion or habitual daily calcium intake between them. Lumbar spine bone mineral content was increased and bone mineral density tended to be higher in those with a greater WC (≥88 cm). These women also had a significantly greater abdominal fat mass, body weight and BMI than those with WC < 88 cm. There was no significant difference in height or lean mass between the groups. Table 3 also presents the measured biochemical variables in women with WC <88 cm and with WC ≥88 cm.

**Table 3:** Anthropometric and biochemical measuresfor the 2 groups of subjects

Anthropometric measurement	Subjects with waist circumference <88 cm; mean (SD) *N* = 16	Subjects with waist circumference ≥88 cm; mean (SD) *N* = 8	*P*
Lumbar spine BMD g/cm2	1.039 (0.212)	1.167 (0.115)	0.06
Lumbar spine BMC g	45.35 (9.95)	55.46 (8.75)	0.011*
Lean mass g	1567 (190)	1540 (253)	0.38
Fat mass g	655 (222)	1071 (132)	0.000**
Height m	1.63 (0.05)	1.63 (0.04)	0.47
Weight kg	62.5 (8.9)	73 (5.6)	0.003*
Waist circumference cm	79 (7)	94 (3)	0.000**
BMI kg/m2	23.57 (2.52)	27.59 (2.38)	0.000**
Fasting glucose mmol/L	4.65 0.49)	5.34 (1.8)	0.08
Total cholesterol mmol/L	5.48 (0.8)	5.98 (0.8)	0.13
Triglycerides mmol/L	0.99 (0.37)	1.24 (0.43)	0.11
HDL mmol/L	1.74 (0.37)	1.6 (0.14)	0.21
LDL mmol/L	3.23 (0.69)	3.6 (1.23)	0.11

*P </=0.01

**P <0.001

**Fig. 2:**
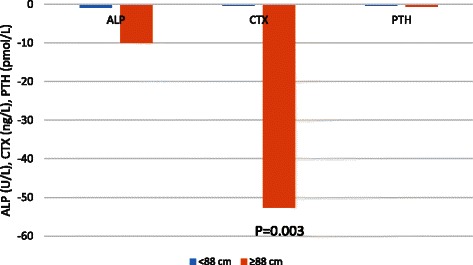
Change in ALP, CTX and PTH (in units as indicated) after the supplemented milk drink in subjects with WC <88 cm and ≥88 cm. The fall in serum CTX was statistically significant (*P* = 0.003)

The changes in measured biochemical variables after the supplemented milk drink in women with and without metabolic syndrome are presented in Table 4. The changes in bone turnover markers ALP and CTX and the calciotropic hormone PTH in each group after the milk drink are depicted in Fig. 2. Women with a larger WC responded to the supplemented milk drink with a significant fall in CTX levels with the fall in ALP tending to significance. There is no evidence that these changes in bone turnover in women with metabolic syndrome are mediated by changes in PTH.

**Table 4:** Changes in biochemical variables after the supplemented milk drink in the 2 groups of subjects

Variable	Change after the supplemented milk drink in women with WC < 88 cm; mean (SD)	Change after the supplemented milk drink in women with WC =/> 88 cm; mean (SD)	*P*
Glucose mmol/L	−0.11 (0.5)	−0.1 (0.36)	0.08
Total cholesterol mmol/L	−0.05 (0.33)	0.09 (0.19)	0.1
PO4 mmol/L	0.03 (0.12)	0.06 (0.04)	0.24
Total Ca mmol/L	0.03 (0.07)	0.02 (0.04)	0.35
Ionised Ca mmol/L	0.02 (0.04)	0.02 (0.03)	0.35
ALP IU/L	−0.9 (5.7)	−10.6 (24.3)	0.07
CTX ng/L	−0.33 (37.46)	−53 (45.2)	0.003*
PTH pmol/L	−0.4 (0.65)	−0.57 (0.66)	0.27

*P <0.005

## Discussion

Weight, abdominal fat mass, BMI and lumbar spine bone mineral content were significantly greater in women whose WC greater than or equal to 88 cm. The lumbar bone density was also higher in these women, but the difference did not achieve statistical significance. There were no significant differences in the biochemical variables or daily dietary calcium intake between the 2 groups of women. Serum 25OHD level was negatively correlated with WC implying that overweight or obese states or metabolic syndrome may be associated with lower vitamin D status.

Following the supplemented milk drink, only ionized Ca changed significantly from baseline. After this drink, the fall in CTX was only significant in women with a WC ≥88 cm and not in those with a WC <88 cm.

Dietary calcium when ingested with a meal including a dairy drink, is influenced by other co-ingested nutrients likely to influence bioavailability and metabolism of calcium. The effect of carbohydrate on bone metabolism is marked and acts through the increase in plasma levels of incretin hormones, particularly glucagon-like peptide 2 (GLP-2) [15]. Osteocalcin, a protein produced by the bone forming osteoblasts is considered to provide a link between energy metabolism and bone [16]. The absence of osteocalcin reduces beta cell proliferation and leads to glucose intolerance and insulin resistance. Osteocalcin is thought to sensitize adipocytes to insulin via adiponectin, thereby improving glucose tolerance. These findings suggest that bone turnover is intimately associated with carbohydrate metabolism and insulin sensitivity or resistance.

In addition protein plays a major role in calcium homeostasis, presumably via IGF-1 [17]. Indeed, medium term studies on dietary calcium intakes show a rise in IGF-1 due to protein in cheese with a concurrent fall in bone turnover markers. Very low protein diets result in hypocalciuria, low fractional calcium absorption and secondary hyperparathyroidism while a high protein diet enhances fractional calcium absorption and consequent urinary calcium excretion [18].

Nutrient intake and caloric intake, among other factors, plays a major role in the development of metabolic syndrome. Metabolic syndrome, due to derangement in various metabolic processes such as insulin resistance, may influence the handling of ingested nutrients. Obesity may protect mammals from osteoporosis, and bone metabolism may indeed be linked to energy metabolism. This link may be the hormones exerting their effects on both bone and energy metabolism under the influence of the hypothalamus [19]. This concept of a gut-brain-bone axis has led to investigations on the effects of nutrients on enteroendocrine cells, and the effect of their secretions (incretins or gut derived hormones) on bone and other tissue. The proposed link between incretins and bone is an evolving concept in nutrient-dependent regulation of bone turnover. The postprandial fall in bone resorption markers and the rise in bone formation markers (albeit to a lesser extent) has been attributed to the effects of incretins GLP-1 mainly, with GLP-2 playing a minor role [20, 21].

The adipocyte derived hormone, leptin is a major regulator of osteoblast function, and inhibit bone formation in vivo [22, 23]. Leptin deficient or leptin receptor deficient mice display an osteopetrotic skeletal phenotype at an early age with high trabecular bone volume [24], however there was a demonstrable decline in the rate of bone formation (BFR) and resorption (BRR). Interestingly, peripheral administration of leptin corrected BFR and BRR demonstrating that leptin was directly involved in regulating bone turnover. In contrast, in humans, low circulating leptin was associated with low bone density [25].

As in any other endocrine pathway, it can be postulated that adipocytes and leptin production in turn, may be under a feedback regulatory control of bone metabolism. Osteocalcin, an osteoblast specific protein, seems a suitable candidate. Indeed, glucose intolerance and greater amounts of visceral fat were noted in Osteocalcin knockout mice (*Osteocalcin*^*−/−*^) [16]. Furthermore, deletion of other genes in osteoblasts in mice led to pancreatic β cell proliferation, a rise in insulin secretion and sensitivity that protected against obesity and diabetes, demonstrating at least the capacity of osteoblasts to contribute to energy metabolism. It follows then that deranged energy metabolism, as in the metabolic syndrome with glucose intolerance and greater visceral fat, may influence bone metabolism. We hypothesis that responses of bone metabolism to dietary calcium may differ between women with and without metabolic syndrome.

## Conclusions

Given that there were no differences in calcium absorption or the rate of urinary calcium excretion between the 2 groups of women, the findings reported here suggest that metabolic syndrome may have influenced the fall of CTX in response to dietary Ca. There was a trend for baseline CTX to be lower in women with higher visceral fat. These findings are consistent with previous proposed theories of obesity protecting against osteoporosis presumably via leptin and hypothalamic feedback, closely associated with energy metabolism [19]. Our data also imply that bone turnover in overweight women or women with metabolic syndrome was more sensitive to dietary calcium intake.
